# Perfect proton selectivity in ion transport through two-dimensional crystals

**DOI:** 10.1038/s41467-019-12314-2

**Published:** 2019-09-18

**Authors:** L. Mogg, S. Zhang, G.-P. Hao, K. Gopinadhan, D. Barry, B. L. Liu, H. M. Cheng, A. K. Geim, M. Lozada-Hidalgo

**Affiliations:** 1 National Graphene Institute, The University of Manchester, Manchester, M13 9PL UK; 2 Department of Physics and Astronomy, The University of Manchester, Manchester, M13 9PL UK; 3 Key Laboratory for Green Chemical Technology of Ministry of Education, Collaborative Innovation Center of Chemical Science and Engineering, School of Chemical Engineering and Technology, Tianjin University, Tianjin, 300072 China; 4 State Key Laboratory of Fine Chemicals, School of Chemical Engineering, Dalian University of Technology, Dalian, 116024 China; 5 Department of Physics, Indian Institute of Technology Gandhinagar, Gandhinagar, Gujarat 382355 India; 6 Shenzhen Graphene Center Tsinghua-Berkeley Shenzhen Institute, Tsinghua University, 1001 Xueyuan Road, Shenzhen, 518055 China

**Keywords:** Materials for energy and catalysis, Graphene, Chemical physics

## Abstract

Defect-free monolayers of graphene and hexagonal boron nitride are surprisingly permeable to thermal protons, despite being completely impenetrable to all gases. It remains untested whether small ions can permeate through the two-dimensional crystals. Here we show that mechanically exfoliated graphene and hexagonal boron nitride exhibit perfect Nernst selectivity such that only protons can permeate through, with no detectable flow of counterions. In the experiments, we use suspended monolayers that have few, if any, atomic-scale defects, as shown by gas permeation tests, and place them to separate reservoirs filled with hydrochloric acid solutions. Protons account for all the electrical current and chloride ions are blocked. This result corroborates the previous conclusion that thermal protons can pierce defect-free two-dimensional crystals. Besides the importance for theoretical developments, our results are also of interest for research on various separation technologies based on two-dimensional materials.

## Abstract (web summary)

Defect-free monolayers of graphene and hexagonal boron nitride are highly permeable to thermal protons, but are impenetrable to gases. Here the authors show that mechanically exfoliated crystals exhibit perfect proton selectivity, corroborating proton transport through the bulk without atomic-scale defects.

## Introduction

Proton transport through two-dimensional (2D) crystals has recently been studied, both experimentally and theoretically^1–9^. As for the experiment, it was found that proton permeation through mechanically exfoliated crystals is thermally activated with energy barriers of ≈0.8 eV for graphene and ≈0.3 eV for monolayer hexagonal boron nitride (hBN)^1^. Further measurements using deuterons, nuclei of the hydrogen isotope deuterium, show that quantum oscillations raise the energy of incoming protons by 0.2 eV^2^. This correction yielded the total barriers of ≈0.5 eV for monolayer hBN and ≈1 eV for graphene. From a theoretical perspective, the latter value is notably lower (by at least 30% but typically a factor of 2) than that found in density-functional calculations for graphene^3–7^. To account for the difference, a recent theory suggests that graphene can be partially hydrogenated during the measurements, which makes its lattice slightly sparser; thus, making it more permeable to protons^8,9^. An alternative explanation put forward attributes the observed proton currents to atomic-scale lattice defects, including vacancies^10,11^. This was argued on the basis of ion-selectivity measurements using chemical-vapor-deposited (CVD) graphene^11^. Indeed, CVD graphene is known to possess a large density of atomic-scale defects that appear during growth^12–14^. Such defects are generally absent in mechanically exfoliated 2D crystals, which was proven conclusively in gas-leak experiments using the so-called nanoballoons^15–17^. Even a single angstrom-sized vacancy per micrometer-size area could be detected in those experiments^16,17^. Whereas it is plausible that vacancies and similar defects played a dominant role in experiments using CVD graphene^10,11^, extrapolation of those results to mechanically exfoliated 2D crystals is unjustifiable. To resolve the controversy, it is crucial to carry out similar ion-selectivity studies using mechanically exfoliated crystals with little or no defects^1,2,15^.

Here we report ion-selectivity measurements using mechanically exfoliated graphene and hBN monolayers. The crystals are found to be perfectly selective with respect to protons. The latter can permeate through the 2D membranes, whereas even such small ions as chlorine are blocked. The results support the previous conclusion^1^ that transport of thermal protons through high-quality graphene and hBN occurs through their bulk and does not involve vacancies and other atomic-scale defects.

## Results

### Device fabrication and characterization

**Fig. 1:**
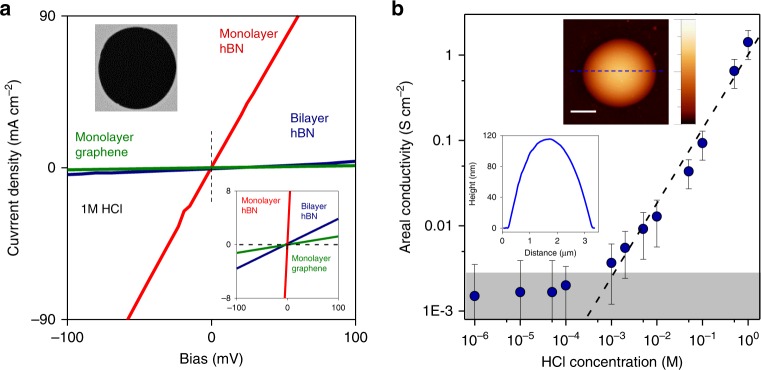
Proton transport through 2D crystals studied using aqueous solutions. **a** Examples of *I*–*V* characteristics for 1 M HCl. Bottom inset: zoom-in. Top inset: electron micrograph of a suspended hBN membrane (aperture diameter, 2 µm). **b** Concentration dependence of the areal conductivity *σ* for monolayer hBN. Gray area indicates our detection limit given by parasitic leakage currents. Error bars: SD from different measurements. Dashed line: best linear fit to the data. Top inset: atomic force microscopy (AFM) height profile of an ‘inflated nanoballoon’. Here, graphene monolayer seals a micrometer-sized cavity containing pressurized Ar. The pressure difference across the membrane makes it to bulge up. Lateral scale bar, 1 μm; color scale, 130 nm. Bottom inset: AFM line trace taken along the blue dotted line in the top inset

The investigated devices were fabricated using monolayer graphene and mono- and bi-layer hBN crystals that were isolated by micromechanical cleavage^18^ (see Methods and Supplementary Fig. 1). The crystals were suspended over microfabricated apertures (2 μm in diameter) etched in free-standing silicon-nitride (SiN) membranes^1^ (Supplementary Fig. 2). A prefabricated polymer washer with a 10 μm-diameter hole was then transferred on top of the crystal so that the hole was aligned with the aperture in the SiN membrane (Supplementary Fig. 2). The assembly was baked at ∼150 °C, to ensure that the washer firmly clamped the 2D crystal to SiN and sealed the crystal edges, to prevent any possible leak along the substrate. In a series of control experiments, we checked that there were no microscopic defects in our exfoliated 2D crystals by employing the approach described in refs. ^15,16^ and previously also used in our experiments^1^. To this end, we made hBN and graphene membranes to cover micrometer-sized cavities etched in an oxidized Si wafer and tested the enclosures for possible gas leaks (see inset Fig. 1b and Supplementary Section ‘Leak tests using nanoballoons’). Even a single vacancy would be detectable in these measurements^16,17^, but neither of the dozens of tested 2D crystals showed such leakage (Supplementary Fig. 3). In contrast, similar devices made from CVD graphene normally exhibited notable gas permeation.

### Ion conductivity measurements

The chips containing the individual 2D membranes (Supplementary Fig. 2) were then used to separate two compartments filled with hydrochloric acid (HCl) at chosen concentrations^19^. Electrical conductance through the membranes was probed using Ag/AgCl electrodes placed inside the compartments. Figure 1a shows the current density *I* as a function of applied voltage *V* for representative devices made from graphene and hBN. The *I*–*V* response was linear, which allowed us to determine the areal conductivity *σ* = *I*/*V*. We found monolayer hBN to be the most conductive of the studied crystals, followed by bilayer hBN and monolayer graphene. For example, using 1 M HCl we found *σ* ≈ 1,000 mS cm^−2^ for monolayer hBN, ≈ 40 mS cm^−2^ for bilayer hBN and ≈ 12 mS cm^−2^ for monolayer graphene. The relative conductivities agree well with those found in the previous studies using Nafion (rather than HCl) as the proton-conducting medium^1^. Thicker crystals (e.g., bilayer graphene) exhibited no discernable conductance, again in agreement with the previous report^1^.

As monolayer hBN exhibited the highest conductivity, we focus our discussion below on this particular 2D material, as it allowed the most accurate ion-selectivity measurements (results for graphene are presented in Supplementary Information). Figure 1b shows *σ* found for hBN at various HCl concentrations (the same concentration was used in both compartments). For concentrations above 1 mM, *σ* increased linearly with HCl concentration. At lower concentrations, the measured current was below our detection limit. The latter was determined by electrical leakage along surfaces of the liquid cell and was of the order of 1 pA as found using control devices with no holes in the SiN membranes^19^. In another control experiment, we used devices with the same SiN aperture but without a 2D crystal. They exhibited conductance at least ~1000 times larger than that for the devices with graphene or hBN crystals covering the aperture (Supplementary Fig. 4). This demonstrates that the reported values of *σ* were limited by the relatively low ion permeation through 2D crystals and the series resistance due to the electrolyte itself could be neglected.

### Proton selectivity

**Fig. 2:**
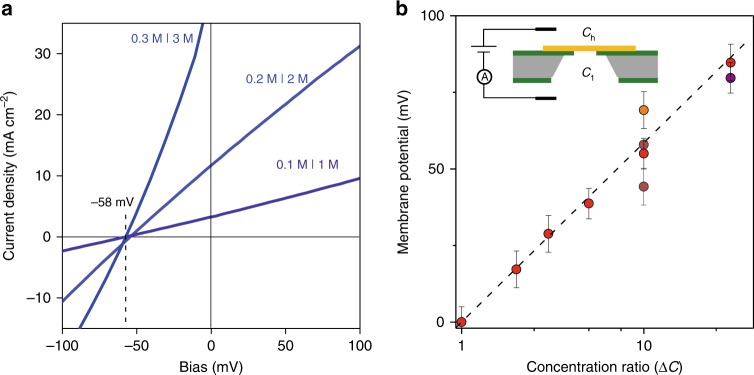
Proton selectivity. **a** Examples of *I*–*V* characteristics for various HCl concentrations across a monolayer hBN membrane at a fixed Δ*C* = 10. The current at zero voltage (intersection with the *y* axis) was always positive. The reversal potential *V*_0_ is given by the intersection of the *I*–*V* curves with the *x* axis and was *V*_0_ ≈ − 58 mV as marked by the dotted line. **b** *V*_0_ for different Δ*C* and four different hBN devices (symbols of different color). Error bars, SD from different measurements. The black line is given by Eq. (1) for *t*_H_ = 1 and *t*_Cl_ = 0. Inset: Schematic of the experimental setup

The measured conductivity could be due to either H^+^ or Cl^−^, or both ions permeating through 2D crystals. For the purpose described in the introduction, it is necessary to determine the fraction of *I* carried by each of these species. Such fractions are usually referred to as transport numbers^20^ (*t*_H_ and *t*_Cl_ for protons and chloride, respectively) and, by definition, they satisfy *t*_H_ + *t*_Cl_ ≡ 1 and the inequality: 0 ≤ both *t*_H_ and *t*_Cl_ ≤ 1. To find their values for our 2D membranes, we used the same setup as in the measurements discussed in Fig. 1 but with different HCl concentrations in the two compartments (inset of Fig. 2b). The concentration gradient drives both H^+^ and Cl^−^ ions towards equilibrium, from the high concentration (*C*_h_) compartment to the low concentration (*C*_l_) one. Therefore, the sign of the total ionic current at zero *V* indicates whether the majority carriers are protons (positive *I*) or chloride ions (negative). Figure 2a shows typical *I*–*V* characteristics for monolayer hBN devices and concentration ratio Δ*C* ≡ *C*_h_/*C*_l_ = 10. Independently of the absolute values of HCl concentrations, the zero *V* current was always positive, proving that the protons dominate ion transport through our membranes. The same behavior was found for graphene devices (Supplementary Fig. 5).

The force pushing ions across the membrane, due to the concentration gradient, can be counteracted by applying voltage *V*. The value *V*_0_ at which the current becomes zero is known as the membrane or reversal potential and is given by the Nernst equation^21^

1 $${\it{V}}_0 = \left( {t_{{\mathrm{{Cl}}}}-t_{\mathrm{{H}}}} \right)\left( {k_{\mathrm{{B}}}T/e} \right)ln\left( {\Delta C} \right) = -\left( {2t_{\mathrm{{H}}}-1} \right)\left( {k_{\mathrm{{B}}}T/e} \right)ln\left( {\Delta C} \right)$$

where *k*_B_ is the Boltzmann constant, *T* is the temperature and *e* is the elementary charge. If one of the transport numbers is unity, the other must be zero and, then, it is said that a membrane displays perfect Nernst selectivity. Figure 2a shows that for Δ*C* = 10, the *I*–*V* curves intersected the *x* -axis at the same *V*, which means that our membranes exhibited *V*_0_ ≈ − 58 mV, regardless of the absolute values of the HCl concentrations. This value is equal to −(*k*_B_*T*/*e*)*ln*(Δ*C* = 10) ≈ − 58 meV at our measurement temperature of ∼20 °C and, therefore, the observation implies *t*_H_ ≈ 1 or, equivalently, that all the ionic current through the membrane is due to proton transport. Within our experimental accuracy, the same perfect selectivity was also found for graphene (Supplementary Fig. 5).

To corroborate the above result and obtain better statistics for the ion selectivity, we carried out similar measurements using different devices and several concentration ratios ranging from Δ*C* = 1 to 30 (Fig. 2b). For all of them, we found membrane potentials consistent with the perfect proton selectivity in Eq. (1). The best fit to the data in Fig. 2b yields *t*_H_ = 0.99 ± 0.02, or *t*_H_ ≈ 1. In control experiments, we verified our experimental approach using porous glass membranes. They allow large concentration gradients but provide no ion selectivity because of large pore sizes. The latter experiments yielded *t*_H_ = 0.81 ± 0.04 (Supplementary Fig. 6), in agreement with the transport numbers known for bulk hydrochloric acid (*t*_H_ ≈ 0.83, *t*_Cl_ ≈ 0.17)^20^.

## Discussion

Finally, it is instructive to compare our results with those obtained previously in conceptually similar experiments but using CVD graphene^11^. The latter was reported to have *σ* ≈ 4 S cm^−2^ at 1 M HCl, in clear disagreement with our experiments for mechanically exfoliated graphene, where *σ* was nearly three orders of magnitude smaller. Furthermore, no current could be detected for 1 mM HCl concentration in our experiments; however, large current densities of ~10 mA cm^−2^ were reported in ref. ^11^ for CVD graphene membranes of the same area. The membrane potential reported for CVD graphene was also different, reaching only ∼8 mV for Δ*C* = 10, or ~7 times smaller than what we found for our devices. All this shows that the ion transport properties of exfoliated 2D crystals are radically different from those of CVD films where atomic-scale defects and, possibly, even macroscopic ones^11^ dominate ion transport. This conclusion is consistent with all the other evidence for intrinsic proton transport through 2D crystals, which was reported previously^1,2^.

In conclusion, our experiments clearly demonstrate that mechanically exfoliated, defect-free 2D crystals allow only proton transport and block even small ions such as chlorine that has one of the smallest hydrated diameters^19^. This provides further support to the view that the activation barriers found for proton transport through high-quality graphene and hBN do not involve vacancies and other atomic-scale defects^1^, a conclusion important for further theory developments (e.g., for the hydrogenation model proposed in refs. ^8,9^). Our results also have implications for the widely discussed use of atomically thin crystals as a novel platform for various separation technologies. In such technologies, selectivity is typically achieved by either perforating nanopores^22–25^ or exploiting those naturally occurring in CVD films^26,27^. The fast permeation of H^+^ through the 2D bulk is usually ignored but can be important for designing and optimizing the membranes’ properties.

## Methods

### Fabrication of 2D membranes

Device fabrication started by isolating atomically thin layers of graphene and hBN from bulk crystals. We used hBN crystals commercially supplied by HQ Graphene. The flake was first identified optically and then characterized using atomic force microscopy and Raman spectroscopy. Supplementary Fig. 1 shows typical characterization data for one of the used hBN crystals. Similar characterization procedures were performed for graphene.

Supplementary Fig. 2 illustrates the device fabrication process. Several lithography, reactive ion etching, and wet-etching steps were performed to obtain a fully suspended SiN membrane with a 2 μm-diameter aperture in the center. The exfoliated 2D crystals were then suspended over the apertures. The crystals were also clamped down to the SiN substrate with a polymer washer. To this end, an SU-8 photo-curable epoxy washer was prefabricated with a 10 μm-diameter hole in the middle and transferred over the devices with the hole and aperture aligned (Supplementary Fig. 2). After the transfer, the seal was hard baked at 150 °C to ensure good adhesion to the SiN substrate.

### Electrical measurements

Devices were clamped with O-rings to separate two reservoirs filled with HCl solutions and Ag/AgCl electrodes were placed inside each reservoir. The *I*–*V* characteristics were measured, applying voltages between typically ±200 mV at sweep rates < 0.1 V min^−1^.

## Supplementary information

Supplementary Information

## Data Availability

The data that support the findings of this study are available from the corresponding author upon request.
