# Supplementary material for: Perfect proton selectivity in ion transport through two-dimensional crystals
Source: Nat Commun. 2019 Sep 18;10:4243. doi: 10.1038/s41467-019-12314-2 (PMC6751181; doi:10.1038/s41467-019-12314-2)
Supplement: Supplementary file 1 — Supplementary Information [file 41467_2019_12314_MOESM1_ESM.pdf]

Perfect proton selectivity in ion transport through two-dimensional crystals

L. Mogg, S. Zhang, G.-P. Hao, K. Gopinadhan, D. Barry, B. L. Liu, H. M. Cheng, A. K. Geim, M. Lozada-Hidalgo

Fabrication procedures

Supplementary Figure 1 shows typical optical, atomic force microscopy and Raman spectroscopy[1] characterization data for one of the used hBN crystals. Similar characterization procedures were performed for graphene. Supplementary Figure 2 illustrates the fabrication flow process.

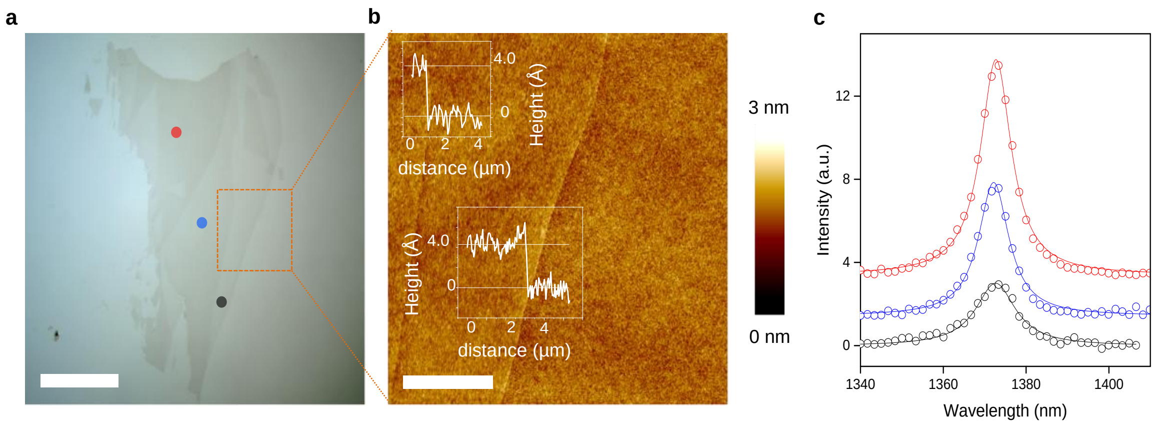

Supplementary Figure 1| Characterization of mono- and few- layer hBN. a, Optical image of a typical hBN flake. Mono-, bi- and tri- layer regions are marked by black, blue and red dots, respectively. Scale bar, 15 µm. **b**, AFM image of the area marked in panel **a** by the red square. The insets show the step heights corresponding to the mono- and bi- layer regions. Scale bar, 4 µm. **c**, Raman spectra from the three areas marked by the dots in panel **a** (color coded). The solid lines are Lorentzian fits.

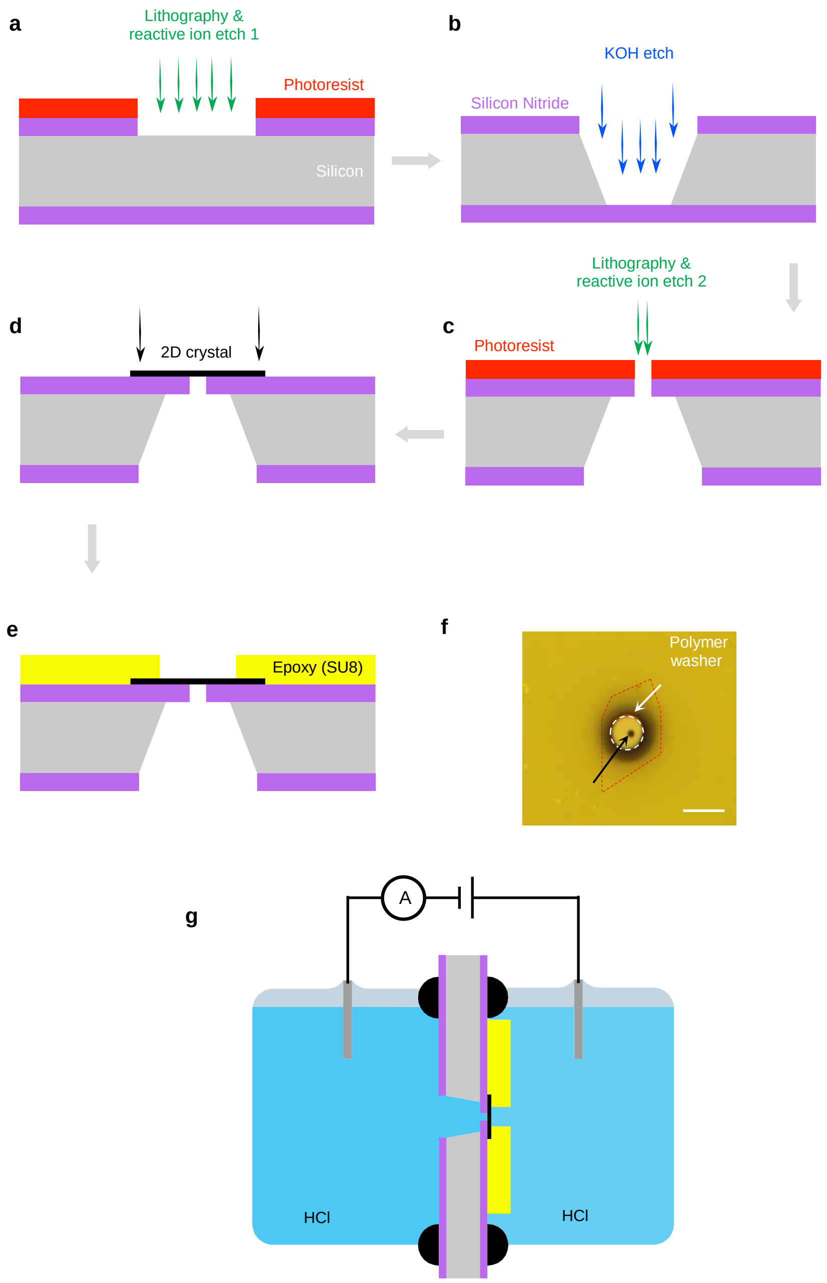

Supplementary Figure 2| Experimental details. **a-e**, Device fabrication flow. Arrows between panels indicate the order in which the different fabrication steps were performed. **f**, Optical micrograph of a final device (top view). The position of the 2D crystal is outlined by the red dotted curve; the circular aperture in SiN is marked with the black arrow; the hole in the polymer washer, with the white dotted circle. Scale bar, 10 µm. **g**, Schematic of our liquid cell. The O-rings used to seal devices are represented with black circles.

Leak tests using nanoballoons

The most sensitive technique to detect microscopic defects in 2D crystals is gas-leak measurements using 'nanoballoons'[2,3]. In such experiments, a small (~1 μm^3) microcavity in an oxidized Si wafer is sealed with a 2D crystal membrane and then filled with a chosen gas (typically, Ar) pressurized above 1 bar[2,3]. The pressure difference between the gas inside and outside the microcavity causes the 2D

membrane to bulge upwards (top inset Supplementary Figure 3). It is possible to monitor changes in the gas pressure inside the microcavity by measuring the membrane deflection using AFM. In the absence of atomic-scale defects, the gas slowly leaks along the silicon oxide layer until the pressure inside and outside the chamber is equalized, a process that typically takes many hours. However, in the presence of even a single angstrom-sized defect (such as a vacancy), the pressure inside the microcavity equalizes typically in seconds[3,4].

To check that our membranes are defect-free, we carried out the above gas-leak experiments following the approach of refs. [[2,3]]. To this end, we etched microcavities in a Si/SiO_2 wafer and sealed them with monolayer graphene. The microcavities were pressurized by placing the devices inside a 'charging' chamber filled with Ar at 2 bar. After several days, the devices were then taken out of the charging chamber and their height profile was measured with AFM. Supplementary Figure 3 shows typical results found for dozens of the membrane devices that were studied. The membranes were found to bulge upwards and the Ar leak rate was found to be ~10^3 atoms per second, in agreement with permeability of the Si oxide layer[2]. Next, in control experiments, we intentionally introduced atomic scale vacancies by mild ultra-violet etch[3]. This procedure yields a defect density so low that it cannot be detected using Raman spectroscopy. Nevertheless, we found that the resulting nanoballoons did not inflate at all, even after leaving them in the charging chamber for over a month. This is consistent with rapid gas effusion through the 2D membranes such that angstrom-sized defects lead to their deflation within seconds, beyond time resolution of our approach[3,4]. The described experiments show that our mechanically-exfoliated crystals were defect-free, in agreement with the conclusions reached in refs. [[3,4]] for similar graphene devices.

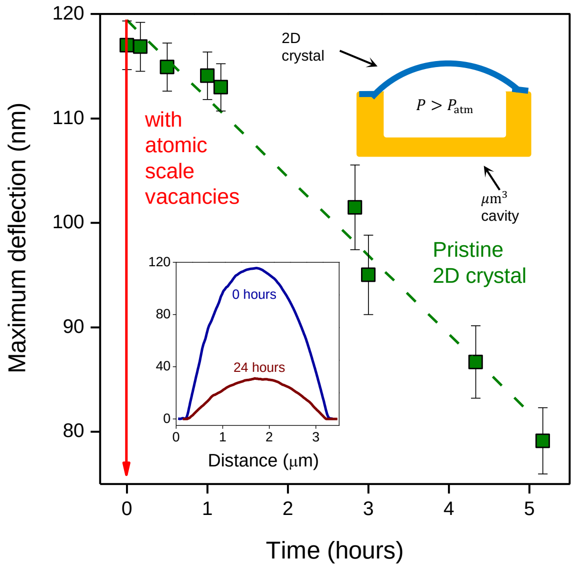

Supplementary Figure 3I Leak tests using nanoballoons. Maximum membrane deflection as a function of time. The data point at time zero hours corresponds to the first measurement after the device was taken out of the charging chamber. It normally took us only several minutes before the first data point was recorded. The red arrow indicates that in the presence of a few atomic-scale defects, we did not observe any bulging at all. Top inset: Schematic of our nanoballoons. Bottom inset: AFM traces taken through the center of an inflated nanoballoon at different times after taking it out of the charging chamber.

Characterization of electrical measurements setup

To characterize our setup, we first determined typical leakage currents, in the absence of any proton conductive path. This was done in two different ways. First, a SiN substrate without an aperture was used to separate two HCl solution reservoirs. Second, a suspended 2D membrane device was used to separate two reservoirs filled with deionized water. In both cases only minute currents of the order of 1 pA were detected. This shows that electrical leakage provided little contribution to the obtained *I-V* characteristics of our 2D-membrane devices. Next, we characterized the maximum possible conductance through our apertures at a given HCl concentration. To this end, we measured devices in which the apertures in SiN were not covered with a 2D crystal (referred to as 'bare aperture devices'). Supplementary Figure 4 shows that σ of such devices scaled linearly with electrolyte concentration. Importantly, we found that for all concentrations, σ of bare-aperture devices was $\gtrsim$1000 times larger than for those with a 2D-crystal membrane.

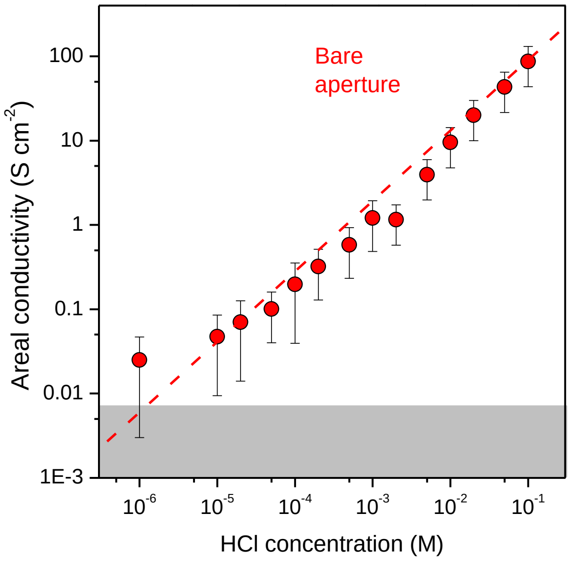

Supplementary Figure 4| Conductance of bare-aperture devices. Their σ as a function of HCl concentration. Dotted line: Best linear fit to the data. The grey area indicates our detection limit determined by pA-range leakage currents.

Membrane potential measurements

To measure the membrane potential for our membrane devices, they were placed to separate two reservoirs filled with HCl solutions at different concentrations. The membrane potential was measured by recording *I-V* characteristics and finding their intersection with the x-axis. Such intersection is known as the zero current or cell potential (V_{cell}) and has two components: the redox potential (V_{redox}) and the membrane potential (V_o):

$$V_{cell} = V_{redox} + V_o \qquad (1)$$

The redox potential appears due the electrodes' material and is independent of the studied membrane. Its value is well known for Ag/AgCl electrodes. For this reason, it is customary to remove this fixed contribution and report only V_0. We followed this convention. Nevertheless, to double-check

this contribution, we also measured our devices using reference electrodes, instead of Ag/AgCl ones[5]. If using the reference electrodes, we indeed found $V_{cell} = V_0$, as expected.

Selectivity of graphene devices

The proton conductance through graphene membranes is at least ~50 times lower than that for monolayer hBN. For this reason, parasitic capacitive contributions from the setup become significant and induce notable errors in the membrane potential measurements. To minimize this problem, we fabricated a device with many (nine) apertures (each of 2 µm in diameter) and then covered all nine with one large mechanically-exfoliated graphene monolayer. This was possible with graphene because, unlike hBN, it can be mechanically exfoliated into crystals of up to hundreds of microns across. Supplementary Figure 5 shows *I-V* characteristics for this device when it was used to separate two HCl solutions at the concentration ratio $\Delta C = 10$. Hysteresis in the *I-V* curve was much smaller than for individual 2 µm apertures but still contributed towards the uncertainty in determining V_0, which was somewhat larger than that for our typical hBN devices (Supplementary Figure 5). We obtained V_o = - 55±9 mV, which within the uncertainty corresponds to the perfect selectivity for protons.

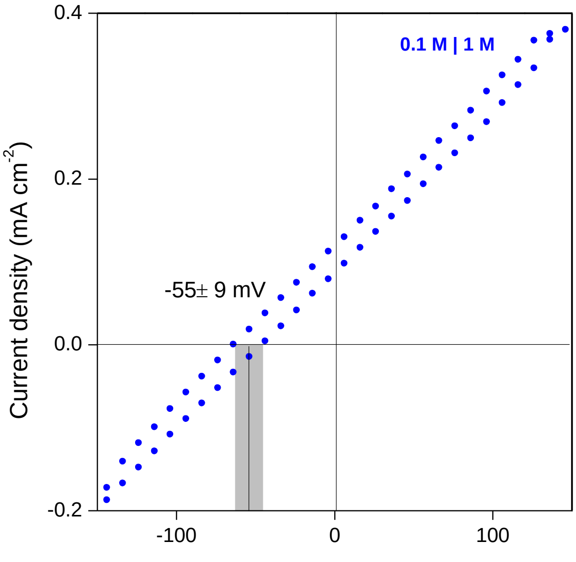

Supplementary Figure 5| Proton selectivity for graphene. *I-V* characteristics of a graphene-membrane device that separated two reservoirs with a concentration gradient of 10. The uncertainty in determining V_0 is marked by the grey rectangle.

Bulk transport numbers for HCl

As a reference, we carried out similar measurements of the membrane potential using a porous glass membrane. Supplementary Figure 6 shows the values of V_0 extracted from these experiments.

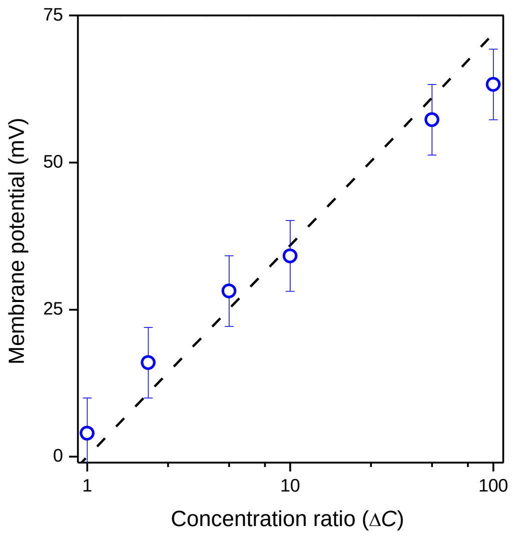

Supplementary Figure 6 | Reference measurements of the membrane potential for porous glass. Symbols: Our experimental data. The black line is given by Eq. (1) and the literature values t_H = 0.83 and t_{Cl} = 0.17 for bulk hydrochloric acid[6].

Supplementary references

1. Gorbachev, R. V. *et al.* Hunting for monolayer boron nitride: Optical and raman signatures. *Small* **7**, 465–468 (2011).
2. Bunch, J. S. *et al.* Impermeable atomic membranes from graphene sheets. *Nano Lett.* **8**, 2458–62 (2008).
3. Koenig, S. P., Wang, L., Pellegrino, J. & Bunch, J. S. Selective molecular sieving through porous graphene. *Nat. Nanotechnol.* **7**, 728–732 (2012).
4. Wang, L. *et al.* Molecular valves for controlling gas phase transport made from discrete ångström-sized pores in graphene. *Nat. Nanotechnol.* **10**, 785–790 (2015).
5. Gopinadhan, K. *et al.* Complete steric exclusion of ions and proton transport through confined monolayer water. *Science* (2019). doi:10.1126/science.aau6771
6. Bard, A. J. & Faulkner, L. R. *Electrochemical Methods*. (Wiley, 2001).
